# Targeting LncRNA‐Vof16: A Novel Therapeutic Strategy for Neuropathic Pain Relief

**DOI:** 10.1111/cns.70241

**Published:** 2025-02-21

**Authors:** Xiuying He, David H. Mauki, Xiaoming Zhao, Songyu Dai, Huisi Yang, Yuexiang Zheng, Qingjie Xia, Rurong Wang, Tinghua Wang

**Affiliations:** ^1^ Department of Anesthesiology, West China Hospital Sichuan University Chengdu China; ^2^ Institute of Neurological Disease, West China Hospital Sichuan University Chengdu China; ^3^ State Key Laboratory of Biotherapy, West China Hospital Sichuan University Chengdu China; ^4^ Department of Basic Medicine, Medical School Kunming University of Science and Technology Kunming China; ^5^ School of Integrated Traditional Chinese and Western Medicine Southwest Medical University Luzhou China

**Keywords:** hyperalgesia, LncRNA‐Vof16, neuropathic pain, peripheral nerve injury, spared nerve injury

## Abstract

**Aims:**

Neuropathic pain (NP) is a debilitating condition characterized by chronic pain resulting from nerve damage or lesion. Despite the ongoing efforts of clinically defining NP, its distinctive mechanisms that lead to various NP phenotypes remain unresolved.

**Methods:**

Using a spared nerve injury (SNI) model, we investigated the mechanisms underlying the development of NP caused by injury in the peripheral nerves. With CRISPR‐Cas9‐mediated knockout and virus‐mediated overexpression strategies, we investigated the role of LncRNA Vof16 (abbreviated as Vof16) during SNI‐induced NP.

**Results:**

Our results revealed that SNI led to the downregulation of Vof16 expression in spinal dorsal horn (SDH) of lumbar enlargement. This was evidently confirmed when we disrupted the expression of Vof16 in SNI rats of which we observed exacerbation of hyperalgesia; while overexpressing it alleviated the pain.

**Conclusion:**

Our findings suggest that Vof16 plays a crucial role in maintaining normal sensory function in healthy states and a protective shield against NP following peripheral nerve injury. We therefore propose Vof16 as a new therapeutic target for alleviating NP.

## Graphical abstract

Graphical abstract showed that loss‐of‐function of LncRNA‐Vof16 (abbreviated as Vof16) aggravates neuronal hyperexcitability and hyperalgesia induced by spared nerve injury. On the contrary, gain‐of‐function of Vof16 holds the opposite and protective effects.

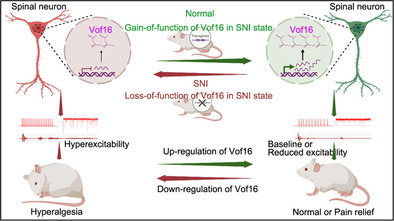

## 1 Introduction

Neuropathic pain (NP) is a significant public health issue resulting from lesions or diseases of the somatosensory system. It is characterized by chronic spontaneous pain, hyperalgesia, and allodynia [1, 2] and is often perceived as a refractory pain syndrome [3, 4, 5]. Growing evidence suggests that central sensitization, driven by neuronal hyperexcitability, plays a crucial role in the development of NP [6, 7]. Therefore, targeting neuronal hyperexcitability could fundamentally address the therapeutic challenges posed by NP. However, the underlying mechanisms of neuronal hyperexcitabilty remain incompletely understood, highlighting the need for extensive exploration in this field to identify new molecular targets for drug development aimed at treating NP.

Long noncoding RNAs (lncRNAs) are transcripts comprised of more than 200 nucleotide bases [8]. Recent evidence has implicated lncRNA in a variety of pathological processes, including carcinoma, neurological disorders, and NP [8, 9, 10, 11]. One notable lncRNA, designated as ischemia‐related factor Vof‐16, is an lncRNA Vof16 with a full sequence of 2108 nucleotides [12]. Michihisa Tohda and his colleagues first reported that Vof16 is closely associated with cognitive disorders resulting from chronic ischemia [12]. Furthermore, Vof16 has been found to be upregulated in the cortex and hippocampus of rats subjected to hypoxic–ischemic (HI) cerebral injury [13, 14], as well as in the damaged spinal cord after spinal cord injury (SCI) [15, 16]. However, disrupting Vof16 accelerated the recovery of HI or SCI‐induced nerve injury and neurobehavioral dysfunction, especially motor function [14, 15, 16]. Notably, other studies have indicated a negative correlation between Vof16 expression and the activity of hippocampal neurons in streptozotocin‐treated diabetic rats [17]. Taken together, Vof16 is highly relevant to neurological diseases and plays a crucial role in regulating neuronal activity. However, the extent to which Vof16 modulates sensory function, neuronal excitability, and hyperalgesia induced by peripheral nerve injury is not fully understood.

The purpose of this study was, therefore, to investigate the role of Vof16 in sensory function and nociceptive behaviors associated with neuropathic pain resulting from peripheral nerve injury. We reported that Vof16 expression was downregulated in SNI model elevating spinal neuronal hyperexcitability and hyperalgesia in rats, and its disruption aggravated the nociceptive symptoms. However, we observed that its overexpression exerted opposite effects, resulting in pain relief. Our findings suggested that Vof16 might act as an endogenous pain inhibitor and neuromodulator in the context of neuropathic pain.

## 2 Methods

### 2.1 Animals

Male Sprague–Dawley (SD) rats, aged 2–3 months and weighing 200–250 g, were housed in a standard 12‐h light–dark cycle with access to water and food ad libitum. All procedures were approved by the Animal Ethics Committee of West China Hospital of Sichuan University and conducted in accordance with the Animal Research: Reporting of in vivo experiments (*ARRIVE*) guidelines.

### 2.2 SNI Model

We used the SNI model to investigate the mechanisms underlying neuropathic pain following peripheral nerve injury. The SNI model was adopted from Decosterd and Woolf [18]. Briefly, rats were anesthetized with 2% isoflurane and exposed the branches of left sciatic nerve. Subsequently, the tibial and common peroneal nerves were ligated with 5‐0 silk sutures and cut, while sparing the sural nerve intact. Notably, the sciatic nerve on the contralateral side on the right was only exposed without injuring any branches.

### 2.3 Behavioral Tests

To investigate the pain induced by SNI, we employed behavioral tests to assess mechanical and thermal hyperalgesia. We used the Von Frey test, a widely accepted method for measuring pain in laboratory animals, to assess mechanical hyperalgesia [19]. Prior to testing, rats were acclimatized for 15 min. Then, a series of Von Frey filaments (0.4, 0.6, 1.0, 1.4, 2.0, 4.0, 6.0, 8.0, 10.0, 15.0, 26.0 g) were applied to the plantar surface of their hind paws according to the up‐and‐down method described by Dixon [20]. Finally, we calculated the 50% mechanical withdrawal threshold (in grams) using Dixon's formula [21] to quantify reflexive responses following SNI‐induced pain.

In addition, we evaluated thermal hyperalgesia in SNI rats using a plantar device (Woodland Hills IITC Life Science, USA). Briefly, rats were placed on a plantar device, and a heat stimulus was applied to the plantar surface of the rat's paw, causing the animal to withdraw its paw as a response. The time it took for the rat to withdraw its paw (latency) was recorded, with each paw tested three times with an interval of 10 min. To prevent tissue damage, we set a cut‐off time of 30 s for each test. This allowed us to assess thermal hyperalgesia by measuring the reduced latency to withdrawal in response to heat stimulation, which indicates an increased sensitivity to heat stimuli, a characteristic of neuropathic pain.

### 2.4 Spinal Cord Slice Preparation and Whole Cell Patch‐Clamp Recording

After anesthetized with 2% isoflurane, rats were transcardially perfused with cutting solution (in mM: 230 Sucrose, 26 NaHCO_3_, 2.5 KCl, 1.25 NaH_2_PO_4_, 0.5 CaCl_2_, 10 MgSO_4_, 10 D‐Glucose; pH 7.4, 290–305 mOsm/L, 95% O_2_ and 5% CO_2_). The spinal lumbar enlargements were removed immediately, fixed in agarose gel (3%), and coronally sectioned by a vibratome (VT1000 A, Leica) in ice‐cold artificial cerebrospinal fluid (aCSF) (in mM: 125 NaCl, 3 KCl, 26 NaHCO_3_, 1.25 NaH_2_PO_4_, 2 CaCl_2_, 1 MgCl_2_, 10 D‐Glucose; pH 7.4, 290–305 mOsm/L, 95% O_2_, and 5% CO_2_). The spinal cord slices (350 μm thick) were incubated in preoxygenated aCSF at 35°C for 60 min and then maintained at room temperature. After incubation, the spinal cord slices were mounted in a recording chamber (perfused with preoxygenated aCSF at ~2 mL/min).

The whole cell patch‐clamp recording was performed on the neurons in lamina I–II of spinal lumbar enlargement. Electrophysiological recordings were sampled at 20 kHz and filtered at 10 kHz at room temperature using a Sutter DIPA amplifier linked to a computer running Sutterpatch software (Sutter, USA). Current‐clamp mode was used to record neural intrinsic excitability with a pipette internal solution comprised as follows (in mM): 135 K‐gluconate, 0.5 CaCl_2_, 2 MgCl_2_, 5 EDTA, 5 HEPES, and 5 Mg‐ATP (PH = 7.3 adjusted with KOH). To record the current threshold for evoking action potential (rheobase) and the neuronal firing rate and behavior, the currents were delivered into the neurons from −80 to 120 pA (10 pA step) and from −80 to 320 pA (20 pA step), respectively. As previously described [22, 23], the neuronal firing behaviors were divided into five patterns here, including no firing, delayed firing, phasic firing, tonic firing, and depolarizing block. Resting membrane potential (RMP) was recorded under I = 0 condition prior to current injection. Voltage‐clamp mode (holding at −70 mV) was applied for spontaneous excitatory postsynaptic current (sEPSC) recording with a pipette containing modified internal solution (the above plus 1 μM bicuculline and 1 μM strychnine). After filled with intracellular solution, the impedance of the patch pipettes was 4–7 MΩ. The series resistance did not exceed 20 MΩ.

### 2.5 In Vivo Extracellular Recording of Spinal WDR Neurons

WDR neurons, located at lamina III–VI of SDH, are associated with the transmission of nociceptive information [24], which respond to mechanical and thermal stimuli from various types of Aδ and C fibers [25, 26]. With in vivo extracellular recording, we detected the activity of WDR neurons during NP following SNI. The experimental procedures referred to previous studies [27, 28]. Briefly, the anesthetized animals were fixed in a stereotaxic apparatus (RWD, China). T13 to L1 laminectomy was performed to expose L3–L5 spinal cord and remove the dura. Bipolar silver electrodes were placed on and transcutaneously electrically stimulated the plantar skin innervated by sciatic nerves [28]. Platinum recording electrodes with an impedance of 2–5 MΩ were positioned by stereotaxic apparatus and placed in L3–L5 SDH (approximately 1 mm lateral to the midline). The extracellular electrical activity of WDR neurons was recorded at a depth of 250–1000 μm (that means the lamina III–VI), and the same position was recorded three times with an interval of 10 min. The stimulation intensity was 1.5× the C‐fiber response threshold. These electrical signals were recorded and amplified using MP150 system (BIOPAC, USA). The field potential and C‐fiber evoked response of WDR neurons were extracted by AcqKnowledge software (BIOPAC, USA). Here, the C‐fiber evoked response was located between 300 and 800 ms of each generated signal.

### 2.6 Statistical Analysis

Data were expressed as the mean ± standard error of the mean (SEM). At first, Kolmogorov–Smirnov test (K‐S test) and Levene's test were used to evaluate normal distribution and homogeneity of variance of data, respectively. If the data followed a normal distribution and the variance was homogeneous, Student *t*‐test or one‐way analysis of variance (ANOVA; followed by LSD or Tamhane's T2 multiple comparisons tests) were then performed to analyze the data from two or three and above completely randomized design groups, respectively. Paired *t*‐test was used to analyze data from two paired samples (e.g., contralateral and ipsilateral). Two‐way ANOVA (followed by a Bonferroni post hoc test) was applied to the repeated data, such as mechanical hyperalgesia threshold, thermal hyperalgesia threshold, body weight, and so on. If not, the data were analyzed via a non‐parametric equivalent. The exact analysis of each data is presented in the corresponding figure legend. *P* value of < 0.05 was considered to be statistically significant. All statistical analyses were performed using IBM SPSS Statistics V19 package (Armonk, NY, USA).

Additional methods have been included in the Data.

## 3 Results

### 3.1 SNI Induces Downregulation of Vof16 in Spinal Dorsal Horn

**FIGURE 1:**
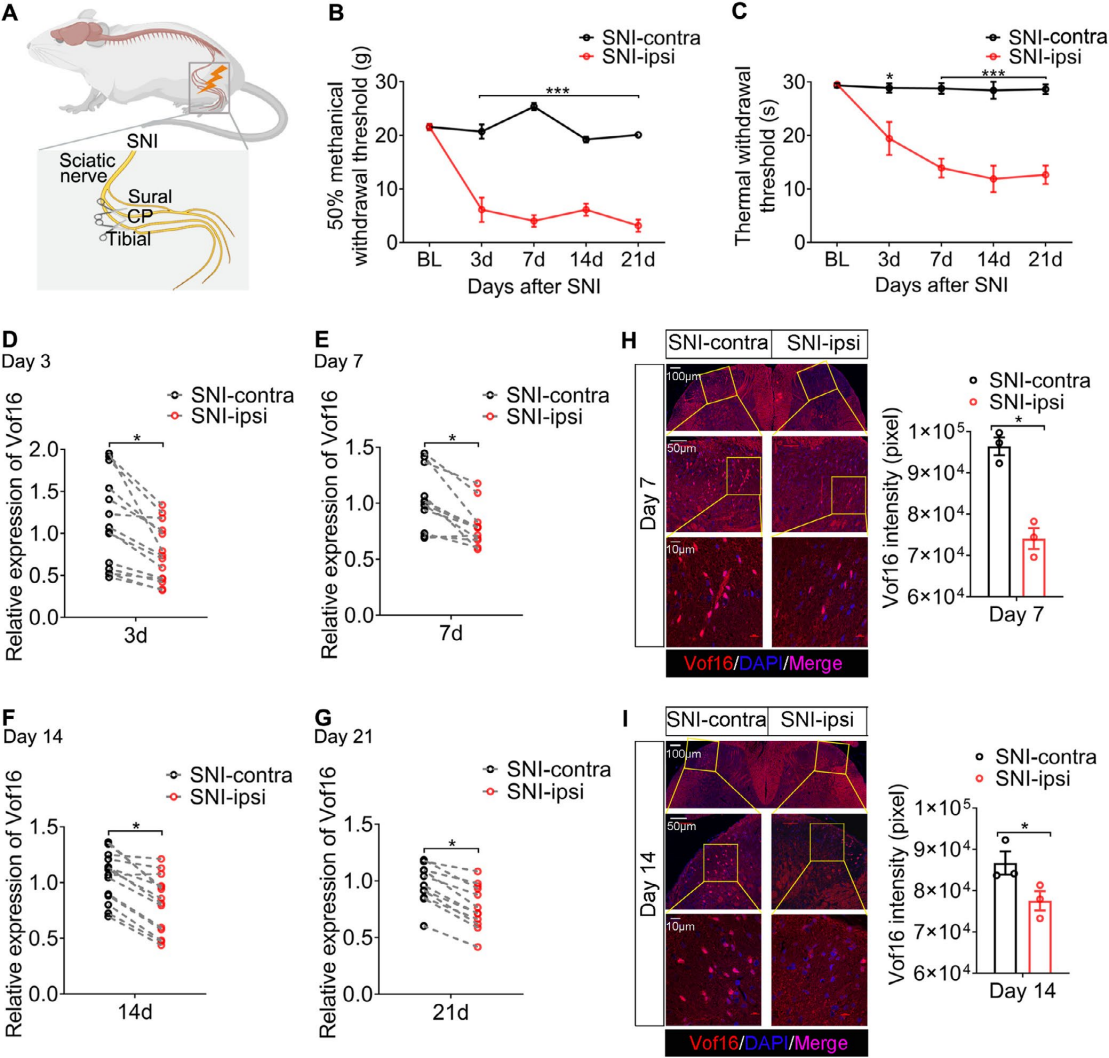
SNI induces downregulation of Vof16 in SDH. (A) Schematic of SNI model in WT SD rats. (B, C) SNI‐induced mechanical (B) and thermal (C) hyperalgesia in adult WT male rats (*n* = 7–9). (D–G) Vof16 was downregulated at SNI 3 days (D, *n* = 14), 7 days (E, *n* = 12), 14 days (F, *n* = 14), 21 days (G, *n* = 12) detected by RT‐qPCR. (H, I) Fluorescent images of spinal lumbar enlargement sections showed the expression of Vof16 (Red) on day 7 (H) and day 14 (I) after SNI. The images below depict the area shown in the boxes of the upper images. contra, contralateral; ipsi, ipsilateral; BL, baseline. Data are presented as mean ± SEM. *p < 0.05, ***p < 0.001, by two‐way ANOVA (B, *F* = 143.65, degree of freedom (df) = 1; C, *F* = 61.18, df = 1) and/or paired *t*‐test (D, *t* = 4.66, df = 13; E, *t* = 4.77, df = 11; F, *t* = 7.02, df = 13; G, *t* = 5.51, df = 11).

We first developed an NP model by SNI in SD rats (Figure 1A). The behavioral results showed that SNI induced mechanical and thermal hyperalgesia, and the symptoms developed 3 days after SNI and lasted for at least 3 weeks (Figure 1B,C). This illustrated that SNI model successfully imitated the nociceptive behaviors of NP. Through reverse transcription and quantitative polymerase chain reaction (RT‐qPCR), we found that SNI resulted in the downregulation of Vof16 in SDH of lumbar enlargement, which emerged 3 days after SNI and maintained in a low state up to 21 days (Figures 1D–G and S1A). Additionally, fluorescence in situ hybridization (FISH) of Vof16 was performed on spinal lumbar enlargement samples on day 7 and day 14 after SNI. The FISH results were congruent with the data from RT‐qPCR (Figure 1H,I). Apart from SDH, the expression of Vof16 in thenar, prefrontal cortex, hippocampus, heart, liver, spleen, and lung tissues was examined on day 14 after SNI. Strangely, except for a slight decrease in thenar innervated by sciatic nerves, there was no change in Vof16 expression in the other tissues after SNI (Figure S1B–H). Because of the close relation between Vof16 and motor function [14, 15] and the existence of pain‐motor protective neuroreflex [29, 30], the decline of Vof16 in thenar (directly mediating the hind limb retraction or foot lifting) might not be accidental, but an adaptive change. To sum up, the above results corroborated that SNI induced downregulation of Vof16 in SDH, indicating that Vof16 might be linked with hyperalgesia caused by SNI.

### 3.2 Disrupting Vof16 Enhances the Intrinsic Excitability of Spinal Neurons, Although It Does Not Induce Hyperalgesia in Non‐SNI State

**FIGURE 2:**
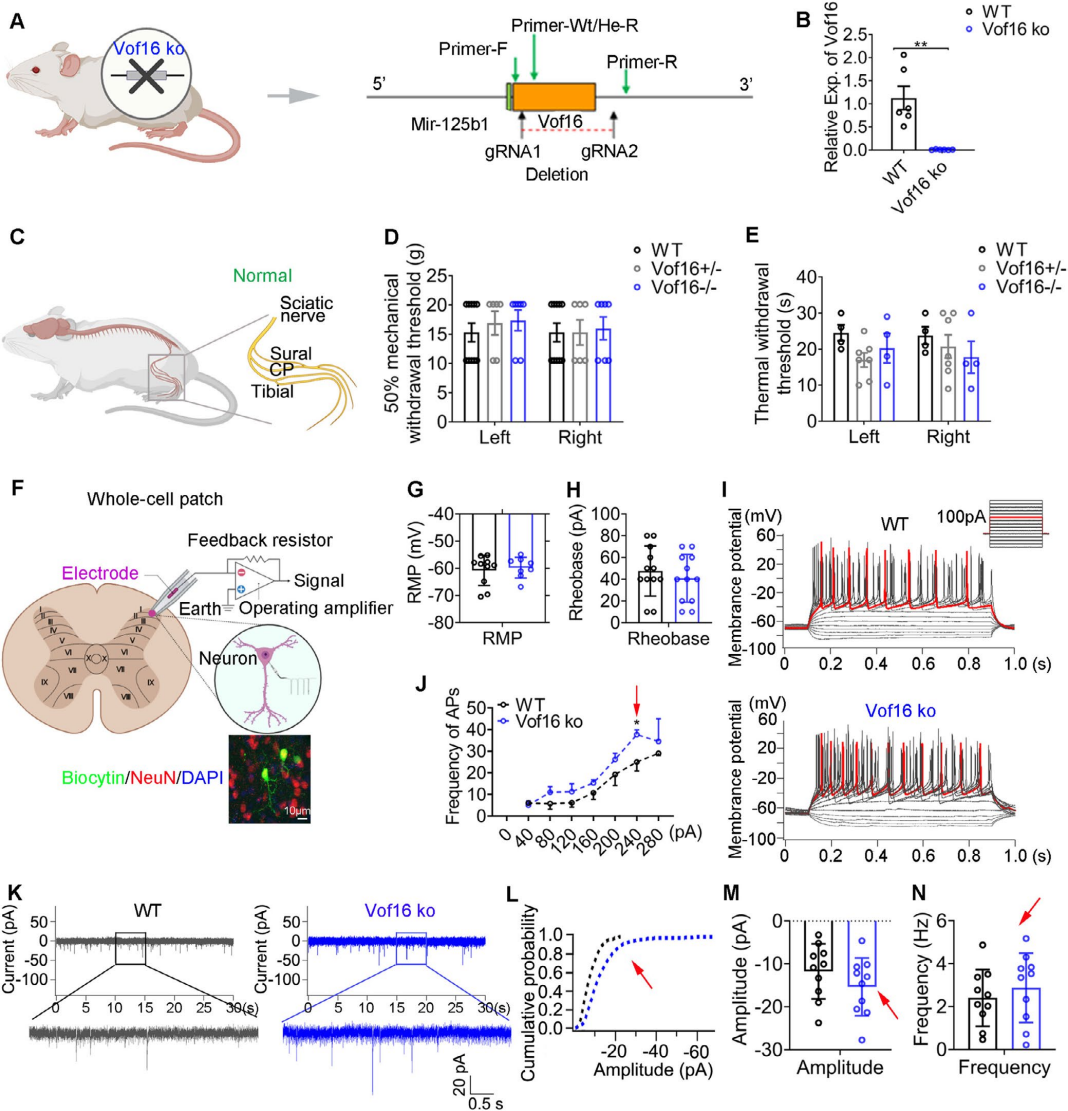
Disrupting Vof16 does not induce hyperalgesia, but enhances the intrinsic excitability of spinal neurons in non‐SNI state. (A) Schematic of knocking out (or disrupting) Vof16 by CRISPR‐Cas9 technique. (B) The expression of Vof16 was extremely low in SDH of Vof16 knockout (ko) rats. (C) Diagram of non‐SNI/normal condition in WT and Vof16 ko rats. (D, E) Vof16 knockdown (Vof16+/−) or ko (Vof16−/−) could not induce mechanical (D, *n* = 7–10) and thermal (E, *n* = 4–7 in the same litter) hyperalgesia in adult male rats. (F) Schematic paradigm of whole‐cell patch recording of neurons in lamina I–II of spinal lumbar enlargement slices (upper). Biocytin was used for marking the recorded neurons (Below). NeuN, the specific marker of neurons. (G, H) The RMP (G) and rheobase (H) were inclined to drop in Vof16 ko neurons (*n* = 10 cells). (I, J) Representative action potential traces (I) and summarized data (J) recorded from WT and Vof16 ko neurons (*n* = 10 cells). Red trace was elicited by 100‐pA current injection. (K–N) Representative current traces (K) and summarized data (the cumulative probability of sEPSC amplitude (L), sEPSC amplitude (M), and frequency (N)) were recorded from WT and Vof16 ko neurons (*n* = 10 cells). Data are presented as mean ± SEM. *p < 0.05, **p < 0.01, by student *t*‐test (B, *t* = 4.41, df = 5; G, *F* = 0.73, df = 22; H, *t* = −0.43, df = 16; M, *t* = 1.22, df = 18; N, *t* = −0.71, df = 18), or/and two‐way ANOVA (D, *F* = 0.19, df = 2; E, *F* = 0.95, df = 2; J, *F* = 7.74, df = 1).

To uncover the role of Vof16, we constructed Vof16 knockout (Vof16 ko) rats by CRISPR‐Cas9 technique (Figures 2A,B and S2A,B). Morphologically, disrupting Vof16 did not hamper the physical and neurological development of SD rats (Figure S2C–H). Above all, the disruption of Vof16 also could not induce hyperalgesia independently in the normal circumstances (i.e., in non‐SNI state; Figure 2C–E).

The electrophysiological characteristics of spinal neurons in lamina I–II of lumbar enlargement slices from wild‐type (WT) and Vof16 ko rats were further recorded by whole‐cell patch clamp (Figure 2F). The intrinsic hallmarks of neurons, including RMP, rheobase, and firing rate of action potential, were evaluated by whole‐cell current‐clamp recordings. Although there was no significant difference in the RMP and rheobase between WT and Vof16 ko neurons, the disruption of Vof16 led to a descending trend in them (Figure 2G,H). Remarkably, disrupting Vof16 improved the firing rate of action potential (*p* = 0.031, a 240‐pA current injection, Figure 2I,J). The synaptic characteristics of neurons were gauged by whole‐cell voltage‐clamp recording of sEPSC. Even though the difference in amplitude and frequency of sEPSC was not statistically significant between Vof16 ko neurons and WT neurons, both of them showed an increasing trend in Vof16 ko neurons (Figure 2K–N).

Taken together, the disruption of Vof16 in non‐SNI condition could slightly enhance the intrinsic excitability of spinal neurons. However, this spontaneous increase in neuronal excitability did not induce hyperalgesia in behavior.

### 3.3 Vof16 Alters the Firing Patterns of Neurons in Spinal Dorsal Horn

**FIGURE 3:**
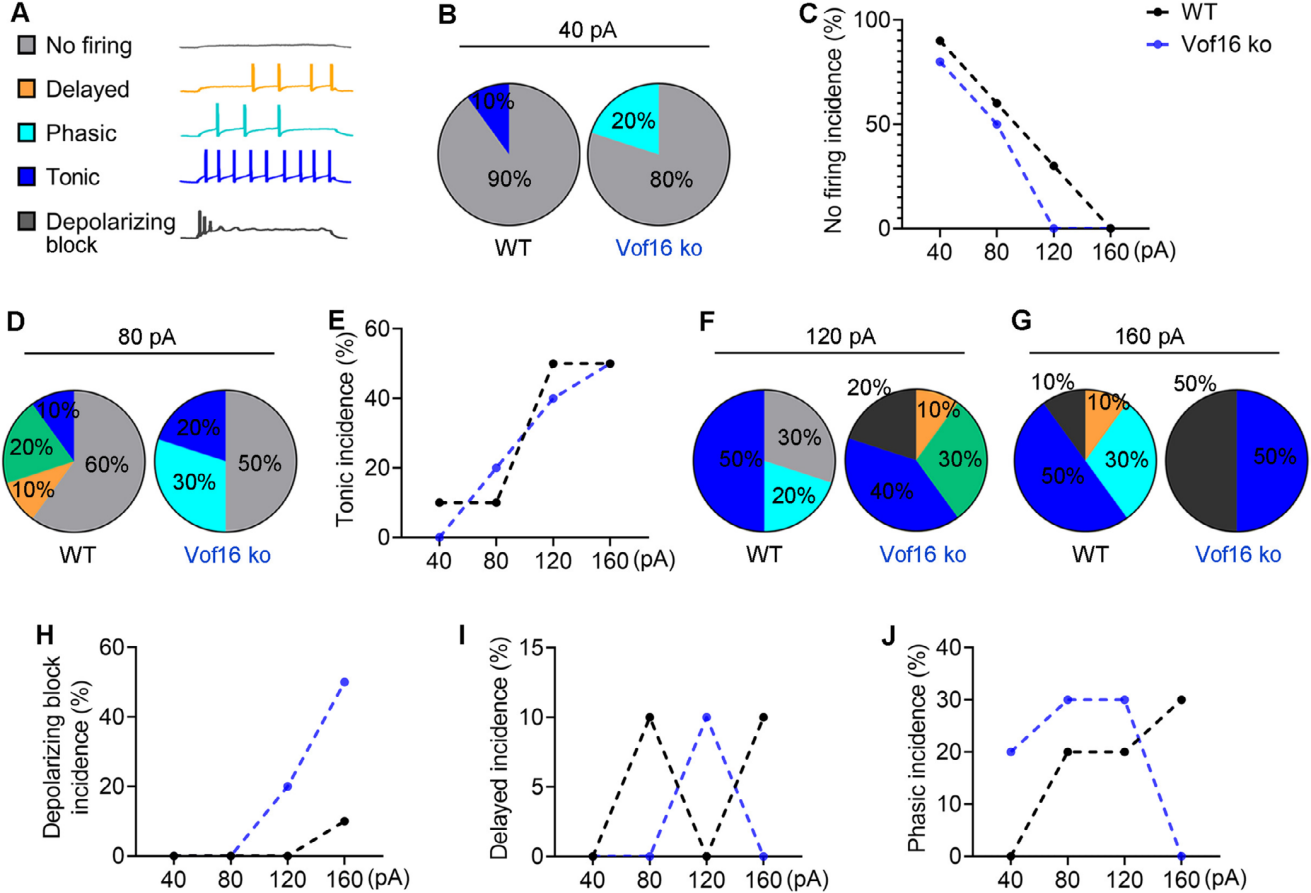
Downregulation of Vof16 alters the firing patterns of neurons in the spinal dorsal horn. (A) The five firing patterns of neurons. (B–J) The incidence of the five different firing patterns under 40‐pA, 80‐pA, 120‐pA, and 160‐pA current injections in WT and Vof16 ko neurons (*n* = 10 cells).

As mentioned above, the firing patterns of neurons consisted of five modes: no firing, delayed firing, phasic firing, tonic firing, and depolarizing block (see Figure 3A). Under distant current stimulation, the firing patterns between Vof16 ko neurons and WT neurons showed significant difference. When responding to small current injection, a 40‐pA current injection was more likely to elicit neuronal firing in Vof16 ko neurons (with 80% of no firing) compared to that in WT neurons (with 90% of no firing; Figure 3B,C). In response to moderate current injection, an 80‐pA current injection could trigger 20% of tonic firing in Vof16 ko neurons, but only 10% of tonic firing in WT neurons (Figure 3D,E). Once larger currents (e.g.,120‐pA or 160‐pA) were input, Vof16 ko neurons were more inclined to depolarizing block (with 20% and 50% in 120‐pA and 160‐pA injection, respectively) in comparison with WT neurons (with 0% and 10% in 120‐pA and 160‐pA injection, respectively; Figure 3F–H). However, we noted that, under different current injections, the incidence of delayed firing and phasic firing varied between WT and Vof16 ko neurons (Figure 3I,J). Generally, these findings here extrapolated that Vof16 ko neurons showed a higher likelihood of excitability compared to WT neurons.

### 3.4 Disrupting Vof16 Aggravates Hyperalgesia and Spinal Neuronal Hyperexcitability in SNI State

**FIGURE 4:**
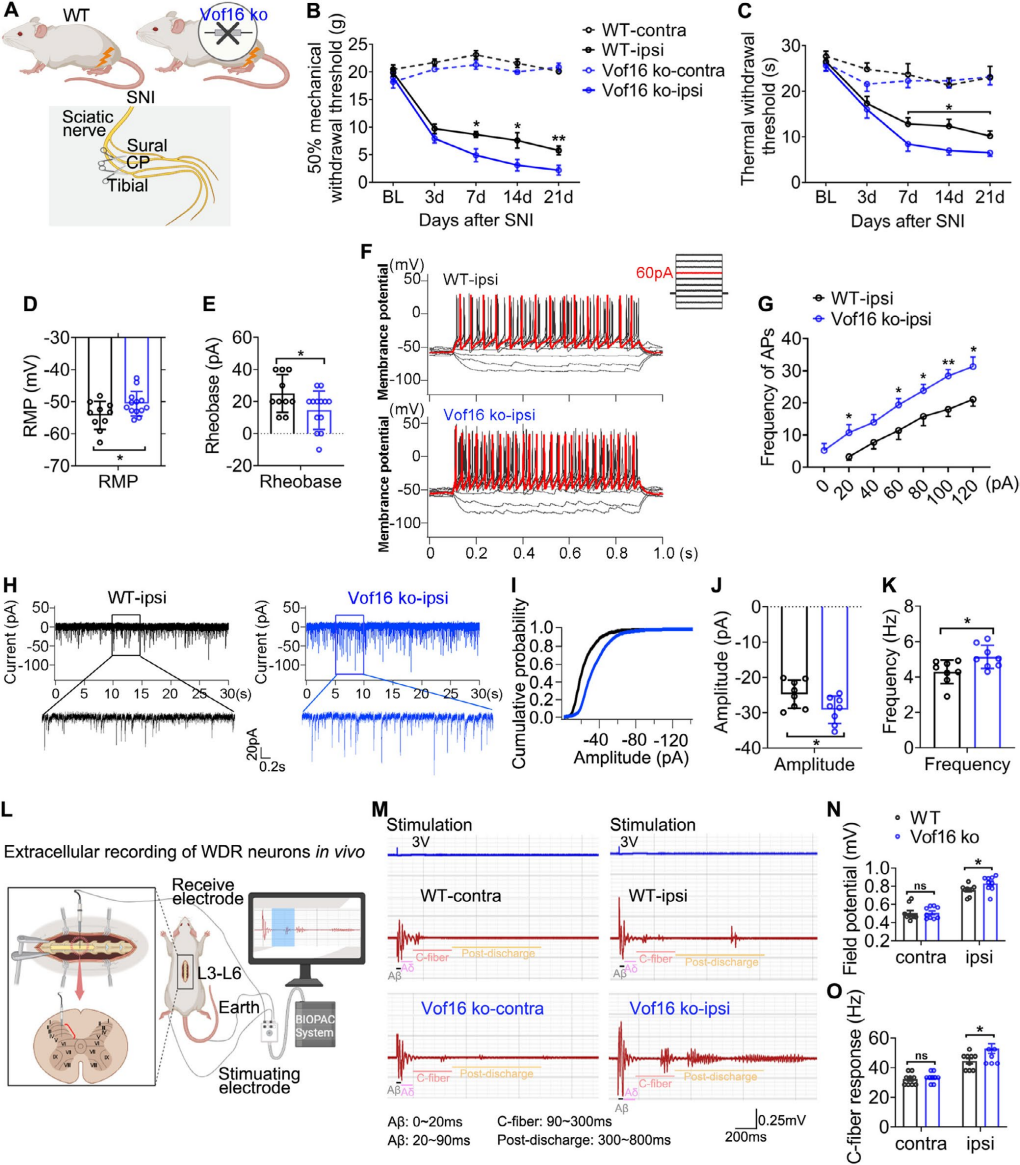
Disrupting Vof16 aggravates hyperalgesia and spinal neuronal excitability in SNI state. (A) Schematic of SNI model in adult male WT and Vof16 ko rats. (B, C) Disrupting Vof16 aggravated mechanical (B, *n* = 8–17) and thermal (C, *n* = 6–8) hyperalgesia in SNI state but not non‐SNI state. (D, E) The RMP (D) and rheobase (E) were dropped in Vof16 ko neurons at SNI 14 days (*n* = 10–13 cells). (F, G) Representative action potential traces (F) and summarized data (G) recorded from WT (*n* = 10 cells) and Vof16 ko (*n* = 13 cells) neurons at SNI 14 days. Red trace was elicited by 60‐pA current injection. (H‐K) Representative current traces (H) and summarized data (the cumulative probability of sEPSC amplitude (I), sEPSC amplitude (J), and frequency (K)) were recorded from WT and Vof16 ko neurons at SNI 14 days (*n* = 8–11 cells). (L) Schematic paradigm of extracellular recording of WDR neurons in vivo. (M–O) Representative traces (M) and summarized data (field potential of WDR neurons (N) and C‐fiber response (O)) were recorded from WT and Vof16 ko rats at SNI 14 days (*n* = 9 data recorded from three rats). Contra, contralateral; ipsi, ipsilateral; BL, baseline. Data are presented as mean ± SEM. ns, not significant; *p < 0.05, **p < 0.01, ***p < 0.001 by two‐way ANOVA (B, *F* = 527.44, df = 3; C, *F* = 38.65, df = 3; G, *F* = 7.10, df = 1; N, *F* = 2.36, df = 1; O, *F* = 4.82, df = 1), or/and student *t*‐test (D, *t* = −2.14, df = 21; E, *t* = 2.08, df = 21; J, *t* = 2.26, df = 14; K, *t* = −2.56, df = 14).

Next, we investigated the effect of blockading the expression of Vof16 in NP following spared nerve injury. To address this issue, we developed an SNI model in both WT and Vof16 ko rats (Figure 4A). As expected, we confirmed that disrupting the expression of Vof16 in SNI state further increased the mechanical and thermal hyperalgesia of rats compared to WT rats (Figure 4B,C). Additionally, the RMP underwent depolarization (Figure 4D), the rheobase substantially declined (Figure 4E), and the firing rate of action potential significantly increased (Figure 4F,G) in Vof16 ko neurons from lamina I to II of the lumbar enlargement comparing with WT neurons in SNI state. As for sEPSC, the amplitude and frequency were boosted in Vof16 ko neurons of lamina I–II (Figure 4H–K). Furthermore, we investigated the excitability and C‐fiber response of WDR neurons in laminae III–VI of WT and Vof16 ko rats by in vivo extracellular recording (Figure 4L). As shown in Figure 4M–O, SNI resulted in elevated neuronal excitability and C‐fiber response in both WT and Vof16 ko WDR neurons (Figure 4M–O). Similarly, the neuronal excitability and C‐fiber response were higher in Vof16 ko WDR neurons than that in WT WDR neurons in SNI state but not non‐SNI state (Figure 4M–O). These results confirmed that the downregulation of Vof16 indeed aggravated hyperalgesia and spinal neuronal hyperexcitability in SNI state.

Previous studies indicated glial cell activation as one of the major features of central sensitization and a contributing factor to hyperalgesia in NP [31, 32]. From this precedent, we also intended to evaluate whether glial cells in the lumbar enlargement regions of the spinal cord were activated as well. Our data revealed that disrupting Vof16 showed no impact on glial cell activation (Figure S3).

### 3.5 Conditional Overexpression of Vof16 in Spinal Neurons Alleviates Hyperalgesia and Neuronal Hyperexcitability Induced by SNI

**FIGURE 5:**
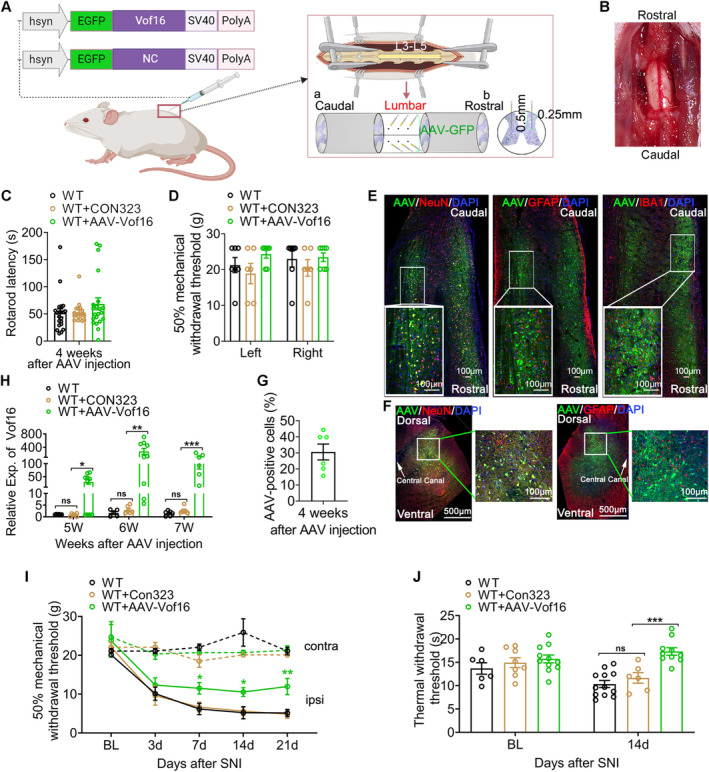
Conditional overexpression of Vof16 in spinal neurons alleviates hyperalgesia induced by SNI. (A) Schematic diagram of intraspinal injection of AAV‐Vof16 and its empty vector (CON323) in spinal lumbar enlargement. (B) The intraspinal injection did not cause visible damage to spinal cord. (C, D) The motor (C) and sensory (D) function of hind limbs were not impaired after AAV intraspinal injection for 4 weeks. (E, F) The immunofluorescence staining of spinal coronal sections (E, 4 weeks after AAV injection) and cross sections (F, 7 weeks after AAV injection) showed that AAV‐Vof16 vector held a respectable neuronal targeted specificity. (G) The percentage of AAV‐positive neurons to total neurons around the injection sites. (H) The expression of Vof16 after AAV injection for 5, 6, and 7 weeks (corresponding to SNI 7, 14, and 21 days). (I, J) Conditional overexpression of Vof16 in spinal neurons relieved mechanical (I, *n* = 8–12) and thermal (J, *n* = 6–12) hyperalgesia in SNI state. BL, baseline; Contra, contralateral; ipsi, ipsilateral. Data are presented as mean ± SEM. ns, not significant; *p < 0.05, **p < 0.01, ***p < 0.001 by one‐way ANOVA (C, *F* = 1.25, df = 2), or/and two‐way ANOVA (D, *F* = 1.61, df = 2; H, *F* = 17.26, df = 2; I, *F* = 16.09, df = 5; J, *F* = 14.62, df = 2). *(Green), WT + AAV‐Vof16 vs. WT + CON323 (in I).

As described above, Vof16 only changed the excitability of neurons, but held no effect on glial cell activation (Figure S3). Therefore, we proposed that neuronal hyperexcitability induced when Vof16 was suppressed during NP might lead to hyperalgesia formation. To further determine the role of Vof16 in neuronal hyperexcitability and NP, we then constructed adeno‐associated virus vector to conditionally overexpress Vof16 gene in spinal neurons (AAV‐Vof16; CON323 is the empty vector), and intraspinally injected it into SDH of lumbar enlargement (Figure 5A). Delightedly, intraspinal injection of AAV vectors with a 33G Hamilton needle at a slow and uniform speed (0.2 μL/min) did not cause visible damage to spinal cord (Figure 5B), or dysfunction in motor and sensory in rats after injection for 4 weeks (Figure 5C,D). Immunofluorescence staining demonstrated that the green fluorescent protein (GFP) carried by AAV vectors was only expressed in spinal neurons, but not in astrocytes and microglia (Figure 5E,F), suggesting that the AAV vectors held a favorable neuronal targeted specificity. As shown in Figure 5G, the percentage of AAV‐positive neurons to total neurons around the injection sites was about 30%. Additionally, as expected, the expression of Vof16 in SDH of lumbar enlargement was substantially elevated in AAV‐Vof16 rats but not CON323 rats after AAV injection for 4 weeks, and this status persisted at least 7 weeks after injection (equivalent to the end of the experiment; Figure 5H). These data indicated that the constructed AAV‐Vof16 vectors could effectively upregulate the expression of Vof16 in spinal neurons.

**FIGURE 6:**
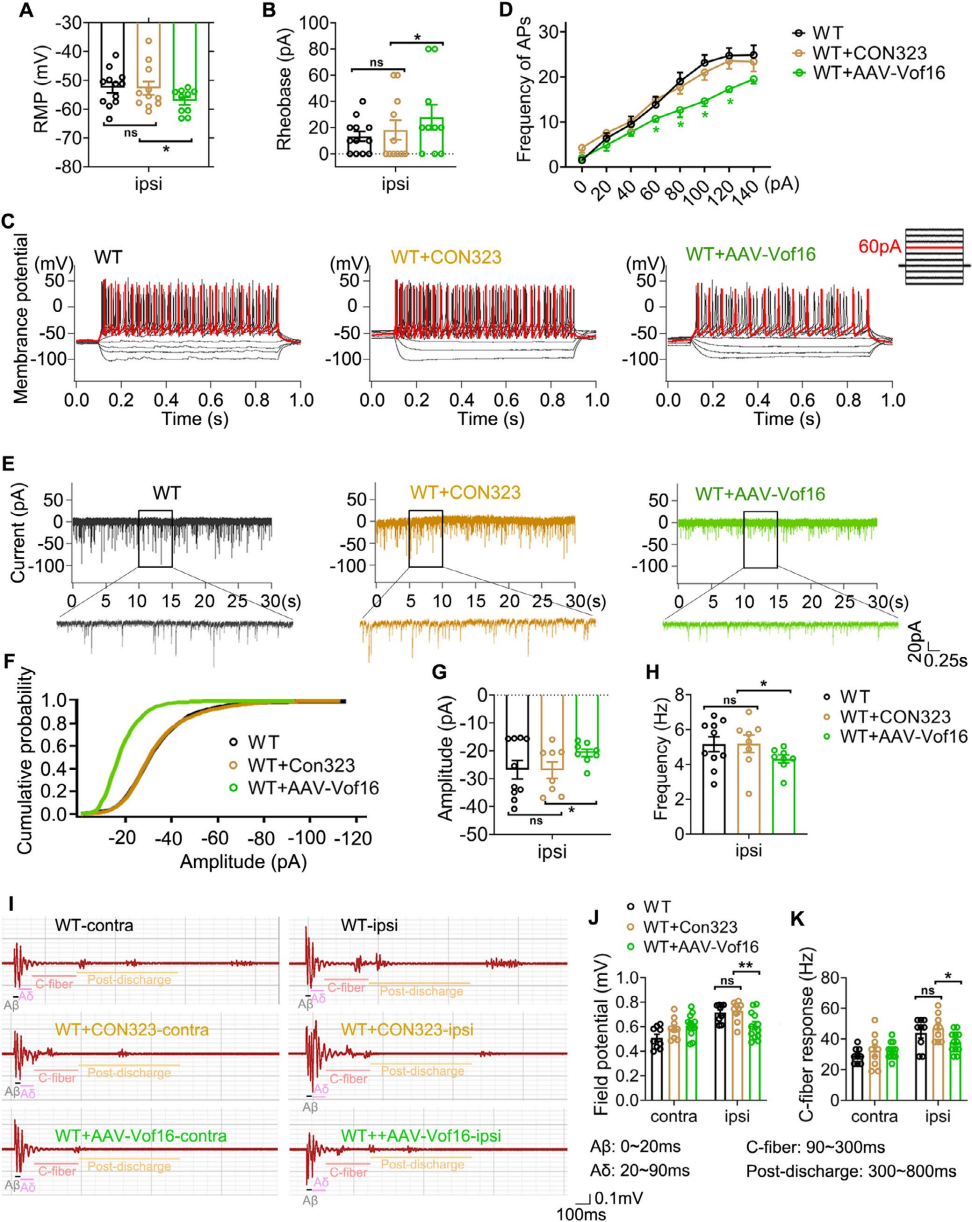
Conditional overexpression of Vof16 in spinal neurons diminishes neuronal hyperexcitability induced by SNI. (A, B) The RMP (A) and rheobase (B) of spinal neurons on the injured side at SNI 14 days (*n* = 9–12 cells). (C, D) Representative action potential traces (C) and summarized data (D) were recorded from WT (*n* = 12 cells), WT + CON323 (*n* = 11 cells), and WT + AAV‐Vof16 (*n* = 10 cells) neurons on the injured side at SNI 14 days. Red trace was elicited by 60‐pA current injection. (E–H) Representative current traces (E) and summarized data (the cumulative probability of sEPSC amplitude (F), sEPSC amplitude (G), and frequency (H)) were recorded in spinal neurons on the injured side at SNI 14 days (*n* = 8–10 cells). (I–K) Representative traces (I) and summarized data (field potential of WDR neurons (J) and C‐fiber response (K)) were recorded at SNI 14 days (*n* = 9 data recorded from three rats). Contra, contralateral; ipsi, ipsilateral. Data are presented as mean ± SEM. ns, not significant; *p < 0.05, **p < 0.01, ***p < 0.001 by one‐way ANOVA (A, *F* = 3.836, df = 2; B, *F* = 2.830, df = 2; G, *F* = 3.242, df = 2; H, *F* = 2.993, df = 2), or/and two‐way ANOVA (D, *F* = 16.368, df = 2; J, *F* = 1.384, df = 2; K, *F* = 1.721, df = 2). *(Green), WT + AAV‐Vof16 vs. WT + CON323 (in D).

Furthermore, the effect of conditionally overexpressing Vof16 on NP induced by SNI was investigated in this study. At first, we excluded the influence of AAV empty vector on the experimental results. As the data were shown, whether in SNI or non‐SNI states, the empty vector (CON323) did not affect not only mechanical and thermal hyperalgesia of WT rats (Figure 5I,J) but also the intrinsic and synaptic characteristics of neurons in lamina I–II of spinal lumbar enlargement (Figures 6A–H and S4). Then, the effect of upregulated Vof16 on hyperalgesia and neuronal excitability was explored. In non‐SNI state, even though conditional overexpression of Vof16 made no impact on mechanical and thermal pain (Figure 5I, upper and 5J, left), it could shrink the intrinsic neuronal excitability (Figure S4). Remarkably, conditional overexpression of Vof16 in spinal neurons attenuated mechanical and thermal hyperalgesia of WT rats in SNI state (Figure 5I,J). In the whole‐cell current patch recording, compared to WT neurons, conditional overexpression of Vof16 impelled spinal neurons to be hyperpolarized (Figure 6A). Moreover, in the spinal neurons with conditional overexpression of Vof16, the rheobase increased significantly (Figure 6B), while the firing rate of action potential was opposite (Figure 6C,D). In the whole‐cell voltage recording, the amplitude and frequency of sEPSC in Vof16‐overexpressed neurons also dwindled significantly in comparison to WT neurons under SNI condition (Figure 6E–H). Additionally, the results of in vivo extracellular recording demonstrated that conditional overexpression of Vof16 abated the field potential and C‐fiber response of WDR neurons in spinal lumbar enlargement in SNI but not non‐SNI states (Figure 6I–K). These results suggested that conditional overexpression of Vof16 in spinal neurons could relieve hyperalgesia and neuronal hyperexcitability induced by SNI.

## 4 Discussion

As mentioned above, Vof16 has been reported to be negatively correlated to neuronal activity and closely associated with nerve injury, especially the damage of motor function [12, 14, 17, 33, 34]. Therefore, we hypothesize that Vof16 might also be involved in regulating nociceptive sensation and neuronal excitability caused by nerve injury. In the present study, we investigated the role of Vof16 in nociceptive sensation induced by SNI. Our data revealed that disrupting Vof16 did not interfere with physical and neurological development, but elevated the excitability of neurons. Nevertheless, this increased susceptibility in neurons could not independently drive the behavioral changes (hyperalgesia) in non‐SNI state. Dramatically, the disruption of Vof16 exacerbated spinal neuronal hyperexcitability and hyperalgesia in SNI state. Conversely, its conditional overexpression in spinal neurons alleviated these nociceptive symptoms.

Our results demonstrated that the downregulation of Vof16 led to the alteration of firing patterns of SDH neurons, which caused Vof16 ko neurons to be more easily excited. This might be a significant reason why the disruption of Vof16 enhanced the excitability of spinal neurons. It was reported that membrane properties determined the firing patterns of neurons [22, 35]. Moreover, the initial membrane potential of neurons was the main factor determining the firing pattern [36]. From depolarized potentials, the neurons fired in a tonic mode [36]. If the holding potential was more negative than the depolarized potential, the characteristic delay to the first action potential occurred [22]. But the delayed firing would turn into tonic mode at more positive potential [22]. When transiently depolarized from the hyperpolarized holding potentials, the neurons fired brief phasic responses [36]. No firing suggested that the membrane potential was hyperpolarized and the injected current was not enough to discharge action potentials. On the contrary, depolarizing block meant that the membrane potential of neurons was more positive than the threshold potential for inducing action potentials and the fast Na^+^ channels were inactivated. In this study, we indeed found that Vof16 could change the initial membrane potential (RMP) of neurons. The RMP of Vof16 ko neurons was more positive than that of WT neurons, while the RMP of neurons with Vof16 overexpression was more negative than that of WT neurons. As it is widely known that the membrane potential is controlled by the ion channels in the cell membranes [37], therefore, the way that Vof16 altered the firing patterns of neurons might be by regulating the ion channels that maintained the membrane potential.

Due to the more positive membrane potential in Vof16 ko neurons, the excitability of these spinal neurons was enhanced, characterized by the increased firing rate of action potentials and the declined rheobase. Then, the excited neurons would increase the action potential and membrane potential‐dependent release of synaptic neurotransmitters to further cause the elevated frequency and amplitude of sEPSC [38, 39, 40, 41]. When the accumulation of excitatory postsynaptic potentials reaches a certain intensity, it stimulates the initial segment of the axon through local electrical currents to generate action potentials, allowing the upstream excitatory signals to continue to be transmitted along nerve fibers [42]. Ultimately, under prolonged stimulation (such as following SNI), the entire sensory conduction system would become sensitized. As reported in this study, the disruption of Vof16 would exacerbate the spinal neuronal hyperexcitability and hyperalgesia in SNI but not non‐SNI states. However, there might be other underlying mechanisms through which Vof16 altered neuronal excitability, necessitating for further investigation.

Spinal dorsal horn represents the first central integration center for nociceptive afferent impulses [43]. According to the Rexed stratification, the dorsal horn region includes lamina I–VI. Peripheral noxious stimuli are first transmitted to the superficial lamina I–II [44], where the interneurons process these afferent information and transmit it upwards [45, 46]. The interneurons in deeper lamina III–VI, such as WDR neurons, could also respond to nociceptive primary afferent inputs [24, 35]. Generally, WDR neurons, located at 250–1000 μm from dorsal surface, respond to mechanical and thermal stimuli from various types of Aδ and C fibers [25, 26], and could identify the small changes in stimulus intensity [47, 48]. Indeed, as the intensity of stimulation expands, the discharge rate of WDR neurons is ramped up [25, 26]. It is also documented that the prolonged hyperexcitability of WDR neurons provoked central sensitization and promoted the response of C‐fiber that transmitted nociceptive stimuli after neuropathy [49, 50]. Based on the ability of encoding stimulus intensity and regulating central sensitization, the role of WDR neurons in SNI‐induced NP was studied in this study. We found that SNI led to the increased field potential and C‐fiber response of WDR neurons in spinal lumbar enlargement. Additionally, disrupting Vof16 further elevated the excitability and C‐fiber response of WDR neurons induced by SNI, while Vof16 overexpression held the opposite effects. These results suggest that Vof16 could not only alter the excitability of neurons in lamina I–II but also modulate the information transmission efficiency of WDR neurons in lamina III–VI.

Our above findings indicate that Vof16 plays a crucial role in maintaining normal neuronal excitability and sensory function in healthy states and a protective shield against NP following peripheral nerve injury (PNI). Therefore, developing methods to elevate the expression of Vof16 during pain would help to reduce the effect and manifestation of pain induced by PNI. Previous studies extrapolated that, the increase in lncRNAs might be caused by the increase in RNA stability, transcriptional activation, and/or other epigenetic modifications [11]. Therefore, we recommend further studies in investigating compounds that could increase RNA stability for the upregulation of Vof16 during NP. Also, identifying and targeting upstream mechanisms (such as transcription factors and DNA methylation) that regulate Vof16 expression could benefit for additional approaches in developing novel therapies for NP. In addition, lncRNAs typically exert their biological functions through physical interactions with regulatory proteins, miRNAs, or other cellular factors [51]. A deeper exploration in the underlying downstream mechanisms that might be associated with the increased neuronal excitability and algesthesia when Vof16 is suppressed during NP would be beneficial for discovering additional molecular targets such as ion channels. Designing drugs that correspond to these downstream targets forms additional strategies for NP therapeutic intervention.

In summary, our study demonstrated that disrupting Vof16 elevated neuropathic pain induced by SNI in rats. However, overexpressing it could alleviate the pain. Despite providing a novel and prominent target for therapy development of NP here, we recommend further studies into probing the underlying mechanisms that Vof16 modulates sensory function and nociceptive behaviors caused by nerve injury.

## Author Contributions

Conceptualization: T.W. and R.W.; Data curation: X.H. and D.H.M.; Formal analysis: X.H., D.H.M. and X.Z.; Funding acquisition: T.W. and X.H.; Investigation: X.H., X.Z., S.D., H.Y., Y.Z. and Q.X.; Methodology: R.W., X.H., D.H.M. and X.Z.; Project administration: T.W., R.W. and Q.X.; Resources: T.W., R.W. and X.H.; Software: X.H., H.Y. and Y.Z.; Supervision: T.W. and R.W.; Validation: X.Z., D.H.M., S.D., H.Y., Y.Z. and Q.X.; Visualization: X.H. and D.H.M.; Writing – original draft: X.H. and X.Z.; Writing – review and editing: T.W., R.W. and D.H.M.

## Conflicts of Interest

The authors declare no conflicts of interest.

## Supporting information


Data S1.

## Data Availability

The data that support the findings of this study are available from the corresponding author upon reasonable request.
