# Correction to “Targeting LncRNA‐Vof16: A Novel Therapeutic Strategy for Neuropathic Pain Relief”

**DOI:** 10.1111/cns.70418

**Published:** 2025-04-28

X. He, D. H. Mauki, X. Zhao, et al., “Targeting LncRNA‐Vof16: A Novel Therapeutic Strategy for Neuropathic Pain Relief,” *CNS Neuroscience & Therapeutics* 31, no. 2 (2025): e70241.

The “Funding” and “Acknowledgments” sections have been updated to acknowledge the valuable contribution of the National Natural Science Foundation of China (Grant No. W2433199).

We apologize for this error.

