# Supplementary material for: Targeting LncRNA‐Vof16: A Novel Therapeutic Strategy for Neuropathic Pain Relief
Source: CNS Neurosci Ther. 2025 Feb 21;31(2):e70241. doi: 10.1111/cns.70241 (PMC11843576; doi:10.1111/cns.70241)
Supplement: Supplementary file 1 — Data S1. [file CNS-31-e70241-s001.docx]

**Targeting LncRNA-Vof16: A Novel Therapeutic Strategy for Neuropathic Pain Relief**

Xiuying He, M.D.^a, b, c, #^, David H. Mauki, Ph.D. ^a, b, #^, Xiaoming Zhao, M.D. ^d^, Songyu Dai, B.D. ^a^, Huisi Yang, M.M. ^e^, Yuexiang Zheng, M.M. ^e^, Qingjie Xia, M.D. ^b^, Rurong Wang, M.D. ^a, *^, Tinghua Wang, M.D. ^a, b, c, e, *^

^a^ Department of Anesthesiology, West China Hospital, Sichuan University, Chengdu, China.

^b^ Institute of Neurological Disease, West China Hospital, Sichuan University, Chengdu, China.

^c^ State Key Laboratory of Biotherapy, West China Hospital, Sichuan University, Chengdu, China.

^d^ Department of Basic Medicine, Medical School, Kunming University of Science and Technology, Kunming, China.

^e^ School of Integrated Traditional Chinese and Western Medicine, Southwest Medical University, Luzhou, China.

*** Corresponding authors:**

**Prof. Dr. Tinghua Wang**, Institute of Neurological Disease, West China Hospital, Sichuan University, Chengdu, Sichuan, 610041, P.R. China; Email: [wangth_email@163.com](mailto:wangth_email@163.com);

**Prof. Dr. Rurong Wang**, Department of Anesthesiology, West China Hospital, Sichuan University, Chengdu, Sichuan, 610041, P.R. China; Email: wangrurong@scu.edu.cn.

#: These authors contributed equally to this work.

**Supplementary methods**

**1. Vof16 knockout SD rats**

Vof16 knockout SD rat was constructed by CRISPR-Cas9 technology in Cyagen Biosciences Inc. Briefly, pUC57-sgRNA vector (referred as sgRNA) was first synthesized based on the knockout target of Vof16. Then injectable Cas9-RNA was obtained by *in vitro* transcription with pRP[CRISPR]-hCas9-U6 plasmid as the templates. Afterwards, a mixture of Cas9-RNA and sgRNA was injected into oosperm by a microinjection platform (Leica, Germony). After a short culture, the oosperm were transplanted into pseudopregnant recipient female rats. Newborn rats were delivered as heterozygotes (F0 generation). All F0 rats were first crossed with wild-type (WT) rats to produce F1 generation to determine if Vof16 deletion could be passed on. Then the heterozygotes from the same litter were crossed again to obtain WT, heterozygous (Vof16+/-) and homozygous (Vof16-/- or Vof16 ko) SD rats. The genotype identification of newborn rats was performed by PCR of genomic DNA with two primer pairs (Table S1).

**2. Rotarod test**

The motor function of hind paws was assessed by a rotarod (ENV-575MA, Med Associates Inc.). Before rotarod test, all rats were acclimatized to the equipment for 5 minutes (min) on the rotating rod at a speed of 4 rpm. Afterwards, the animals underwent a formal 5-minute rotarod test with an accelerating rotating speed from 4 to 40 rpm. The duration of rats on the rotating rod was recorded. Each rat was tested 3 times with an interval of 20 min.

**3. Reverse transcription and quantitative polymerase chain reaction (RT-qPCR)**

Total RNA was extracted according to the instructions of TRIzol reagent (Invitrogen, USA). After the purity of RNA samples were detected by Nanodrop 2000 (Thermo Scientific, USA), qualified RNA was reverse transcribed into cDNA using reverse transcription kit (BioRAD, Germony). Then, a 20 µl reaction system for qPCR was prepared using the cDNA as templates, and the assay was performed on the machine with cycling conditions of 95 °C for 30 seconds (s), 95 °C for 5 s, and 60 °C for 30 s for 40 cycles. The threshold cycle (Ct value) for each sample was recorded. Lastly, β-actin (Actb) was used as an internal reference for semi-quantification of target genes by the 2^-ΔΔCt^ method. All the primers for RT-qPCR were shown in Table S2.

**4. Immunohistochemistry, Fluorescence in situ hybridization (FISH), and Nissl staining**

After deep anesthesia with 2 % isoflurane, rats were transcardially perfused with 0.9 % saline for 5 min, followed by 4 % paraformaldehyde. Then L3 ~ L5 dorsal root ganglion (DRG), spinal cord and sciatic nerves were taken out. After dehydrated in 30 % sucrose solution, the tissues were sectioned (20 μm) with a freezing microtome (CM2800E; Leica, USA).

For immunostaining, the sections were blocked with Ca^2+^/Mg^2+^-free phosphate buffer saline (PBS) containing 5 % sheep serum (Beyotime Biotechnology, China) and 0.3 % Triton X-100 (Sigma-Aldrich, Germony) at 37 °C for 30 min. Then the sections were labeled with primary antibodies (shown in Table S3) at 4 °C overnight. After washing, sections were incubated with secondary antibodies (see Table S3) for 1 ~ 2 hours at 37 °C. DAPI was used for nuclear staining.

For FISH of Vof16, the operations were performed under RNase-free conditions. The tissues were fixed in 4 % paraformaldehyde, permeabilized with 3 ‰ Triton X-100 for 5 min, and then incubated with prehybridization solution (RIBOBIO, China) for 30 min at 37 °C. After that, the samples were hybridized with RNA probes (RIBOBIO, China) overnight at 4 °C. After washing, the cell nuclei were marked by DAPI. All fluorescent images were captured by a Nikon N-SIM-S confocal microscope (Nikon, Japan).

The Nissl staining solution (Beyotime Biotechnology, China) was used for staining neurons. Briefly, the sections were staining with Nissl staining solution for 15 min at 37 °C. Then, the sections were successively washed with 95 % ethanol for about 5 s, and with distilled water twice. Finally, the images were obtained and analyzed by an orthomosaic microscope (SUNNY OPTICAL THCHNOLOGY CO., LTD, China).

**5. Primary culture of spinal and cortical neurons**

Newborn 1-day-old SD rats were decapitated to obtain spinal and cortical neurons. In brief, the spinal cord and cortex were taken out and digested with 0.25 % trypsin at 37 °C for 10 min. The obtained cells were filtered with a disposable sieve (the diameter of the hole was 200 mesh). After centrifugation, cell precipitates were obtained and resuspended with neuron specific medium (neurobasal medium (Gbico, USA): B27 (Invitrogen, USA) = 50:1). Then the cells were kept in an incubator with 5 % CO_2_ and 95 % humidity at 37 °C and the medium was changed every 3 days.

**6. AAV construction and intraspinal injection**

The recombined AAVs (rAAVs) were constructed in BrianVTA (Wuhan, China). In brief, the recombinant plasmid vectors, with Vof16-EGFP sequence and the neuron-specific promoter-hSyn, were constructed and then transfected into AAV-293 cells with pHelper and pAAV-RC. After 2 to 3 days of transfection, the rAAVs were packaged and amplified in AAV-293 cells. After cell lysis, the rAAVs particles were released. The injectable rAAVs (AAV-Vof16 and the empty vectors) were acquired by purification.

The rAAVs were intraspinally injected into SDH of lumbar enlargement with a stereotaxic apparatus. In short, rats were anesthetized with 2 % isoflurane, the L3 ~ L5 spinal cord was exposed, and the spine of rats was fixed to the stereotaxic instrument. The Hamilton microinjector with a 33G needle entered the spinal dorsal horn at an angle of 45° at 1 mm lateral to the midline. There were 6 injection points on the L3 ~ L5 spinal cord, which were divided into two columns on the left and right and three rows on the upper, middle and lower sides with 1 mm of interval. Moreover, each point had two injection depths on the Z axis, including 250 μm and 500 μm. The injection volume of each point was 1 μl (about 2×10^10^ rAAVs) at a speed of 0.2 μl/min, and the needle was kept for 2 min before and after injection. After intraspinal injection, the back skin was sutured and disinfected, and the rats were put back into the feeding cages for resuscitation. SNI model was constructed after AAV injection for 4 weeks.

**Supplementary results**

**1. Disrupting Vof16 does not interfere with physical and neurological development of rats.**

The Vof16 knockout rat was constructed by CRISPR-Cas9 technique. The disrupted gene sequence of Vof16 was shown in Fig S2A. Due to the overlap of the first 500bp of Vof16 gene with miRNA-125b1, only the Vof16 gene sequence after miRNA-125b1 was knocked out to maintain the latter function. Owing to the long sequence of Vof16, the genotype identification of newborn rats was performed by PCR of genomic DNA with two primer pairs (Table S1). Then the genotype of rats was determined as shown in Fig. S2B. We noticed that the body weight and physique of Vof16 knockout (Vof16-/- or Vof16 ko) or knockdown (Vof16+/-, heterozygous) rats were analogous to that of WT rats (Fig. S2C-D), supporting that disrupting Vof16 did not hamper the growth and physical development of SD rats. Moreover, the disruption of Vof16 did not impinge on the number and distribution of sciatic nerve fibers (Fig. S2E) and neurons in dorsal root ganglia (including neurofilament 200 (NF200), isolectin-B4 (IB4) and calcitonin gene related peptide (CGRP) positive neurons; Fig. S2F) and spinal neurons (Fig. S2G). In addition, neither Vof16 knockdown nor Vof16 knockout affected the growth and neurite length of primary cultured spinal and cortical neurons *in vitro* (Fig. S2H). Shortly, disrupting Vof16 did not interfere with physical and neurological development of rats.

**2. Disrupting Vof16 holds no impact on glial cell activation.**

The glial cell activation in lumbar enlargement at 14d and 21d after SNI was evaluated by RT-qPCR and immunofluorescence. The data from immunofluorescence revealed that SNI led to the incremental immunoreactivity of microglia marker-ionized calcium bindingadaptor molecule-1 (Iba-1) and astrocyte marker-glial fibrillary acidic protein (Gfap), but there was no difference between WT and Vof16 ko sections in non-SNI or SNI states (Fig. S3A-F). These results were further confirmed by RT-qPCR (Fig. S3G-N). In a word, disrupting Vof16 held no impact on glial cell activation.

**Supplementary figures**


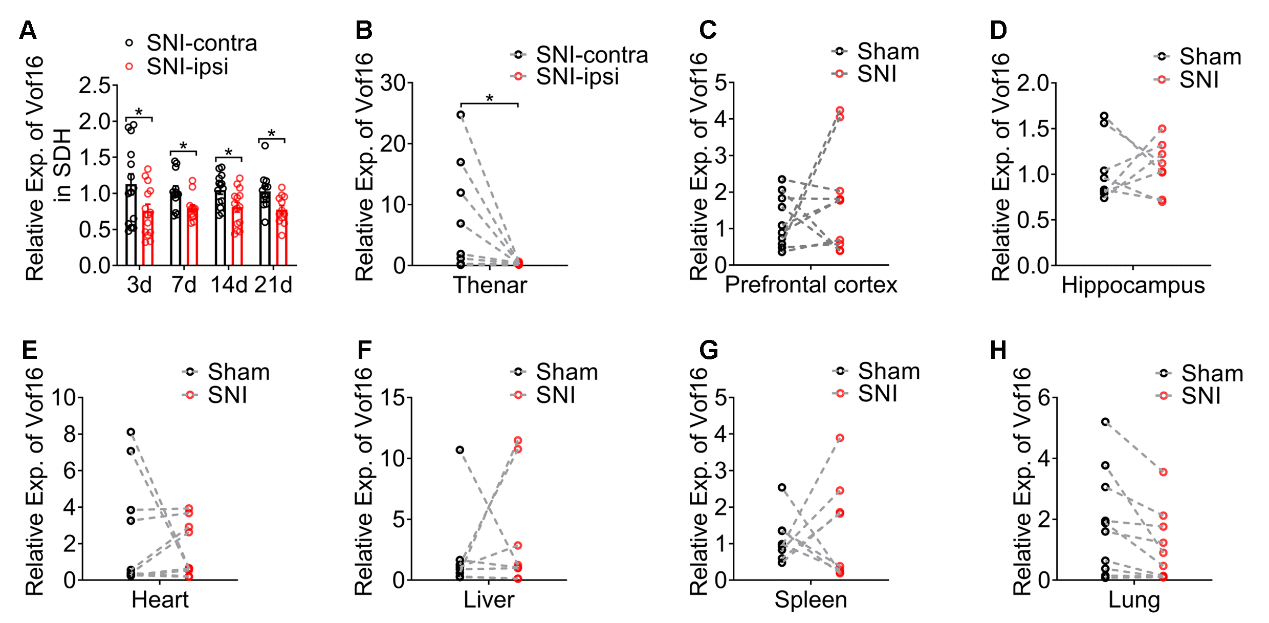


**Fig S1. The expression of Vof16 in SDH, thenar, prefrontal cortex, hippocampus, heart, liver, spleen and lung after SNI.** (A) The Vof16 expression changes over time following SNI. (B-H) The expression of Vof16 in thenar (B), prefrontal cortex (C), hippocampus (D), heart (E), liver (F), spleen (G) and lung (H) at day 14 after SNI. contra, contralateral; ipsi, ipsilateral. * P < 0.05 by two-way ANOVA (A, F= 31.713, df = 1), paired t-test (B, t = 2.223, df = 18) or/and student t-test (C, t = -1.169, df = 18; D, t = -0.202, df = 14; E, t = 0.782, df = 13.177; F, t = -0.712, df = 14; G, t = -0.484, df = 10.056; H, t = 1.269, df = 18).


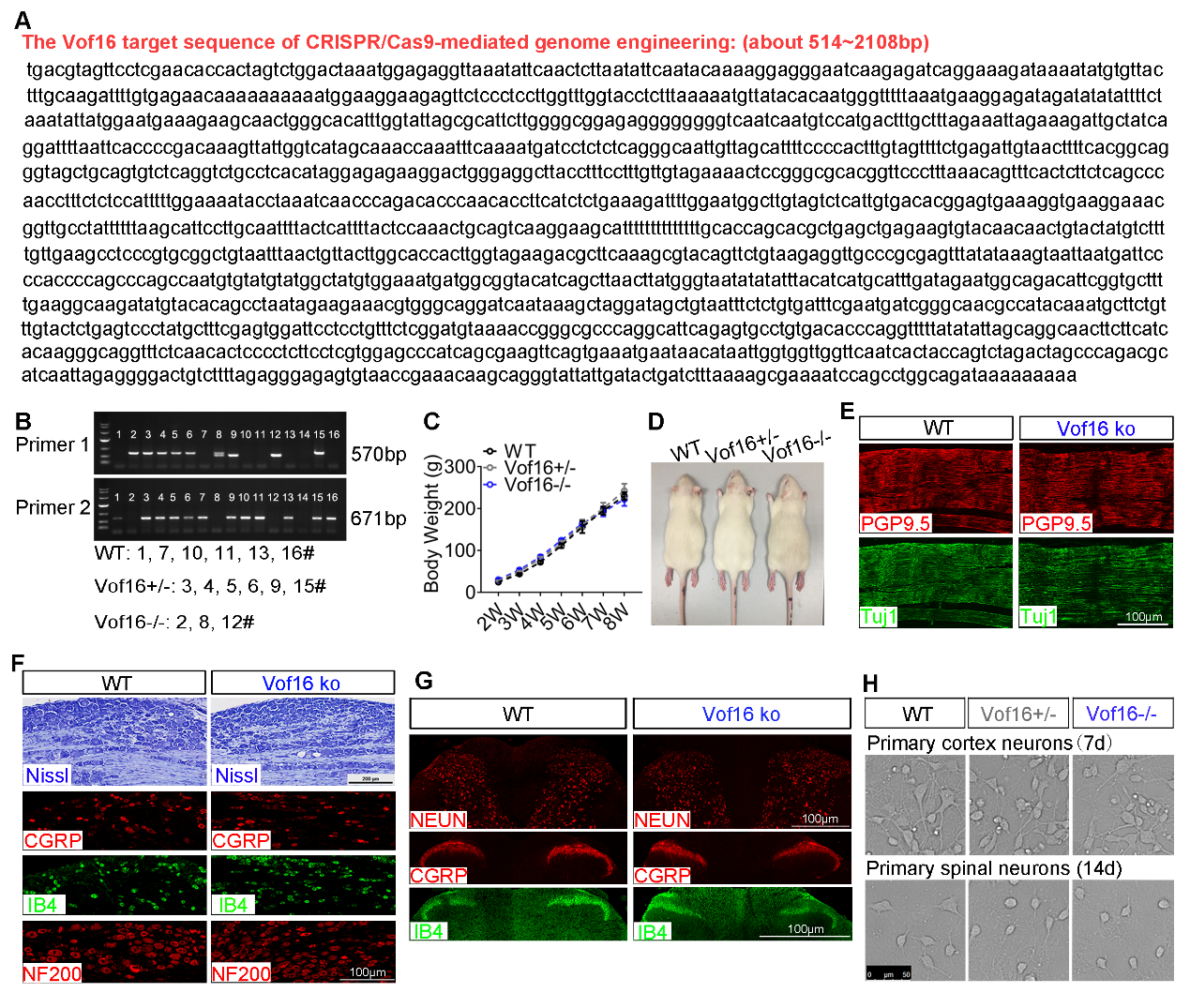


**Fig S2. Disrupting Vof16 does not affect physical and neurological development of rats.** (A) The disrupted gene sequence of Vof16 by CRISPR-Cas9. (B) The results of genotype identification. (C, D) Vof16 knockdown or knockout did not affect the body weight (C, n = 8 ~ 11) and physique (D). (E-G) No difference in number and distribution of sciatic nerve fibers (E), dorsal root ganglion neurons (F) and SDH neurons (G) was observed in WT and Vof16 ko rats. (H) The morphology of primary cultured cortical (7d, uppper) and spinal neurons (14d, below) was unaffected by Vof16 knockdown or knockout. Data are presented as mean ± SEM. Significance was assessed by two-way ANOVA (C, F = 0.965, df = 2).


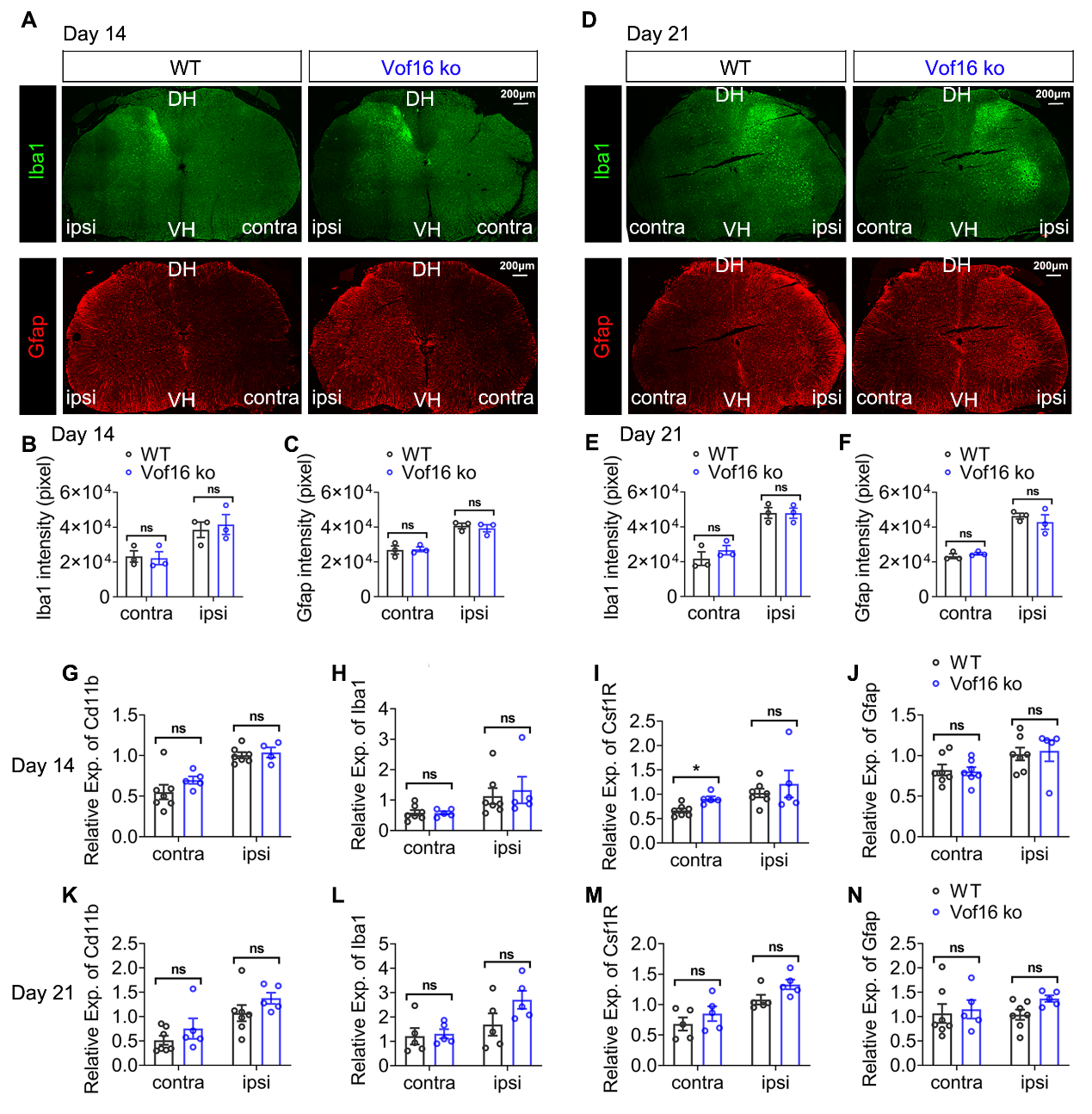


**Fig S3. Disrupting Vof16 holds no effect on glial cell activation.** (A-F) Representative immunofluorescence images (A, D) and summarized data (Iba1 intensity (B, E) and Gfap intensity (C, F)) in spinal lumbar enlargement sections of WT and Vof16 ko rats at SNI 14d and 21d (n = 3 sections from three rats). (G-J) Disrupting Vof16 did not alter the mRNA expression of Cd11b (G, K), Iba1 (H, L), Csf1R (I, M) and Gfap (J, N) in SDH from lumbar enlargement at SNI 14d and 21d (n = 5 ~ 8), in which Cd11b, Iba1 and Csf1R were the markers of microglia activation and Gfap was a marker of astrocytic activation. contra, contralateral; ipsi, ipsilateral; VH, ventral horn; DH, dorsal horn. Data are presented as mean ± SEM. ns, not significant, by two-way ANOVA (B, F = 0.064, df = 1; C, F = 0.140, df = 1; E, F = 0.059, df = 1; F, F = 0.676, df = 1; G, F = 0.935, df = 1; H, F = 0.263, df =1; I, F = 0.376, df =1; J, F = 0.010, df = 1; K, F = 0.829, df = 1; L, F = 5.757, df = 1; M, F = 3.997, df= 1; N, F = 2.478, df = 1).


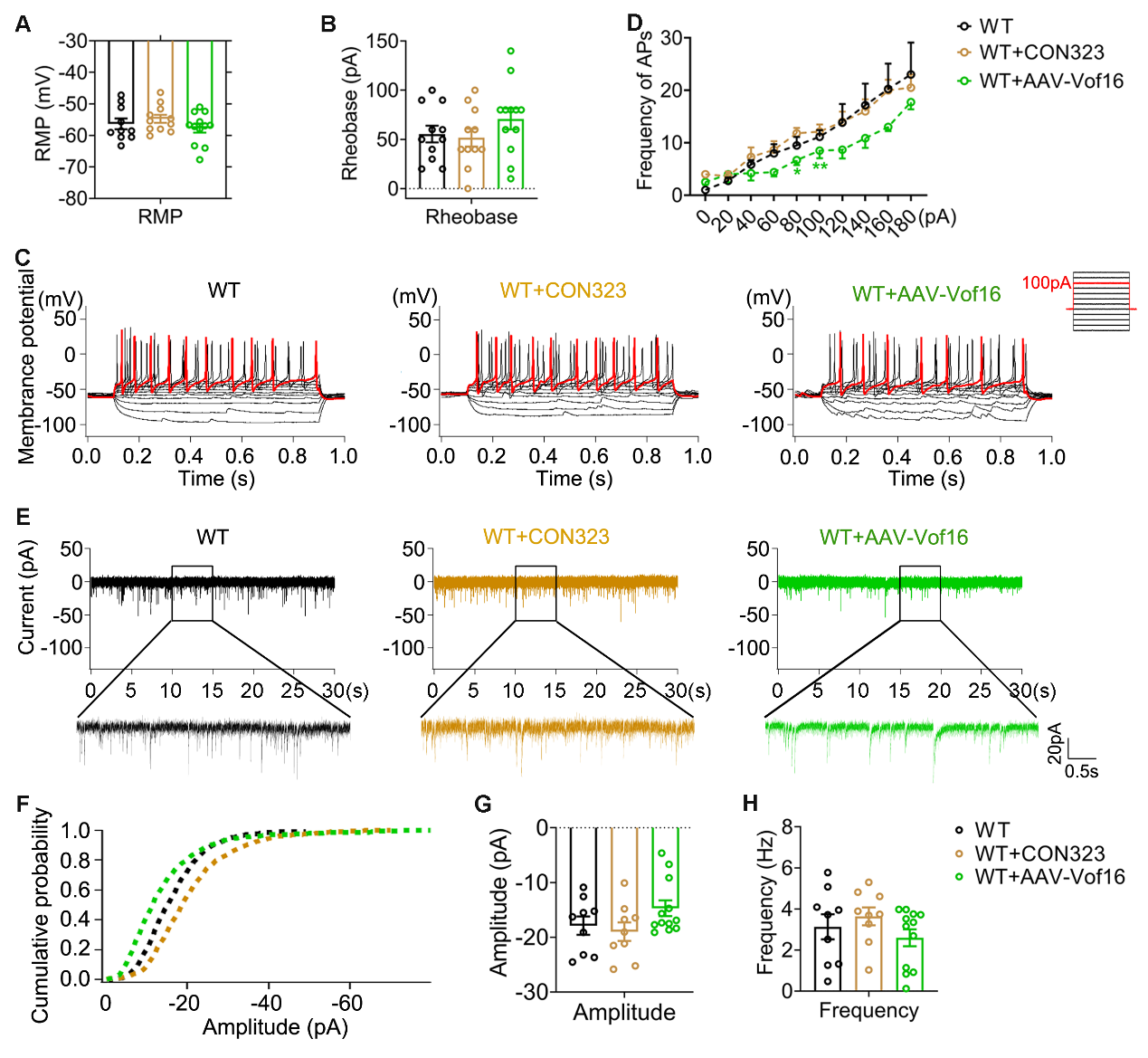


**Fig S4. Conditional overexpression of Vof16 in spinal neurons tends to abating neuronal excitability in non-SNI state.** (A, B) The RMP (A) and rheobase (B) of spinal neurons in lamina I ~ II of lumbar enlargement from Sham rats (n = 11 ~ 12 cells). (C, D) Representative action potential traces (C) and summarized data (D) were recorded from spinal neurons (WT (n = 11 cells), WT+CON323 (n = 11 cells), and WT+AAV-Vof16 (n = 12 cells)) of Sham rats. Red trace was elicited by 100-pA current injection. * (Green), WT+AAV-Vof16 vs WT+CON323 in (D). (E-H) Representative current traces (E) and summarized data (the cumulative probability of sEPSC amplitude (F), sEPSC amplitude (G) and frequency (H)) were recorded in spinal neurons of Sham rats (n = 9 ~ 12 cells). Sham rats: only the left sciatic nerve was exposed and undamaged. Data are presented as mean ± SEM. ns, not significant, * P < 0.05 and ** P < 0.01 by one-way ANOVA (A, F = 1.015, df = 2; B, F = 1.150, df = 2; G, F = 2.841, df = 2; H, F = 1.211, df = 2) or/and two-way ANOVA (D, F = 216.000, df = 2).

**Supplementary tables**

**Table S1. The two primer pairs for genotype identification**

| Primers | Sequences (5' to 3') |
| --- | --- |
| Rat-Vof16-1-Forward | AGTTTGTCCGAGTGATGGGAATACAC |
| Rat-Vof16-1-Reverse | GACTCCGTGAGCCATTTATCATTCTG |
| Rat-Vof16-2-Forward | AGTTTGTCCGAGTGATGGGAATACAC |
| Rat-Vof16-2-Reverse | ATGTGCCCAGTTGCTTCTTTCATTC |

**Table S2. The sequence of all RT-qPCR primers**

| Gene | Forward Primers (5' to 3') | Reverse Primers (5' to 3') |
| --- | --- | --- |
| Vof16 | CCCGACAAAGTTATTGGTCATAGC | AAAGGTAAGCCTCCCAGTCCTTC |
| Cd11b | GACCTTCCAAGAGAATGCAAGT | TGCTGTAGTCACACTGGTAGA |
| Gfap | AGATCGCCACCTACAGGAA | CACGATGTTCCTCTTGAGGT |
| Iba-1 | TCCGAGGAGACGTTCAGTTA | GTTGGCTTCTGGTGTTCTTTG |
| Csf1R | TACCTGTTCACTCCAGTGGT | GTGTAGTTGTTGCCCTCGTAG |
| β-actin | CCGCGAGTACAACCTTCTTG | CGACGAGCGCAGCGATA |

**Table S3. The primary and secondary antibodies for immunohistochemistry**

| Antibodies | Animal origin | Dilutions | Campany | Catalog Number |
| --- | --- | --- | --- | --- |
| Anti-NeuN | Mouse | 1:100 | Abcam | ab279295 |
| Anti-Gfap | Mouse | 1:400 | Proteintech | 60190-1-Ig |
| Anti-Iba1 | Rabbit | 1:100 | Cell Signaling Technology | 17198 |
| Anti-PGP9.5 | Rabbit | 1:100 | Proteintech | 14730-1-AP |
| Anti-Tuj1 | Mouse | 1:500 | Abcam | ab78078 |
| Anti-Isolectin B4 (IB4), FITC conjugated | - | 1:200 | Sigma-Aldrich | L2895 |
| Anti-NF200 | Rabbit | 1:100 | Proteintech | 18934-1-AP |
| Goat anti-Mouse secondary antibody, Cy3 conjugated |  | 1:100 | Jackson ImmunoResearch | 115-165-003 |
| Goat anti-rabbit secondary antibody, Cy3 conjugated |  | 1:100 | Jackson ImmunoResearch | 111-165-003 |
| Goat anti-Mouse secondary antibody, FITC conjugated |  | 1:200 | Abcam | ab150113 |
| Goat anti-rabbit secondary antibody, FITC conjugated |  | 1:100 | Abcam | ab150077 |
